# 1,2,3-Triazole-Substituted Oleanolic Acid Derivatives: Synthesis and Antiproliferative Activity

**DOI:** 10.3390/molecules18077661

**Published:** 2013-07-01

**Authors:** Mariano Walter Pertino, Cecilia Lopez, Cristina Theoduloz, Guillermo Schmeda-Hirschmann

**Affiliations:** 1 Instituto de Química de Recursos Naturales, Universidad de Talca, Casilla 747, Talca, Chile; 2 Facultad de Ciencias de la Salud, Universidad de Talca, Talca, Chile

**Keywords:** oleanolic acid, click chemistry, antiproliferative activity

## Abstract

Hybrid compounds are relevant products when searching for structure-activity relationships of natural products. Starting from the naturally occurring triterpene oleanolic acid, alkyl esters were prepared and treated with different aromatic azides using click chemistry to produce hybrid compounds. Some 18 new oleanolic acid derivatives were synthesized and the structures were confirmed by spectroscopic and spectrometric means. The antiproliferative activity of the new derivatives was evaluated towards normal lung fibroblasts (MRC-5), gastric epithelial adenocarcinoma (AGS), promyelocytic leukemia (HL-60), lung cancer (SK-MES-1) and bladder carcinoma (J82) cells. The alkyne esters **1** and **3** showed activity on all cell lines but without selectivity (19.6–23.1 μM and 14.1–56.2 μ*M*, respectively), their respective methyl esters were inactive. Compounds with a benzene and *p*-anisole attached to the triazole ring, showed no antiproliferative effect. Introduction of a chlorine atom into the benzene ring (compound **9**) elicited a selective effect against AGS cells (IC_50_ value: 8.9 *μ*M). The activity was lost when the COOH function at C-28 was methylated. Better antiproliferative effect was found for compounds **11** and **15** bearing a *p*-toluenesulphonyl group, with values in the range of 10.8–47.1 *μ*M and 11.5–22.2 *μ*M, respectively. The effect, however, was not associated with selectivity.

## 1. Introduction

Terpenes are compounds that present several biological activities. In the last decades, some studies have shown the widespread promise of triterpenes as templates for selected bioactivities. Modifications of oleanolic acid (OA) as well as some closely-related triterpenes such as betulinic acid and dihydrobetulinic acid have led to anti-HIV agents [1], anti-human melanoma compounds [2], anticancer prodrugs [3] and to molecules with antiproliferative and/or cytotoxic effects [4,5]. A review on pentacyclic triterpenes as tools in cancer therapy that includes OA has recently been published [6].

Most of the work on bioactive OA derivatives has been carried out with naturally occurring compounds isolated from plants. Esters or amides at C-3 and/or C-28 were prepared to disclose structure-activity relationships of the products on selected biological targets [4,7,8,9]. However, little has been done on the application of click chemistry techniques to obtain structural diversity starting from OA. Recently, OA derivatives were prepared and assessed for cytotoxic effect using *N*-aryl-*N'*-hydroxyguanidine to prepare C3-esters. Some of the compounds prepared showed strong and selective cytotoxic activity against SMMC-7721 cells [10]. An additional article reported the synthesis and cytotoxic activity of twelve OA derivatives on PC3, A549 and MCF-7 cells. Some of the semisynthetic compounds showed similar or higher cytotoxicity than OA [9].

Click chemistry is a term which describes an efficient 1,3-dipolar cycloaddition reaction between alkynes and azides to obtain 1,4-disubstituted triazoles. Triazoles have been shown to possess desirable features in medicinal chemistry. The triazole are stable to acid and basic hydrolysis and reductive and oxidative conditions, because of their high aromatic stabilization. In addition, this heterocycle has a high dipole moment and might participate actively in hydrogen bond formation as well as in dipole–dipole and π stacking interactions [11]. Last, this compound is relatively resistant to metabolic degradation [12]. For many years, alkylating agents have been studied with regard to cancer chemotherapy, and this has led to the development of many new and more selective alkylating agents including molecules that are based on the triazole moiety [13,14,15,16]. Recently, 1,2,3-triazole have shown antiproliferative properties [17,18]. Wang [19] proposed that planar heteroaromatic triazole derived compounds might lead to a more facile interaction with DNA, proteins, or cells. They assessed abilities for apoptotic induction, using a BGC target cell line, which is a common model for testing general antitumor compound activities and for clarifying molecular mechanisms. Besides, this heterocycle also possesses other activities like cytotoxic [20], anti-HIV [21], antibiotics [22] and bactericidal effects [23].

Click chemistry of natural products has acquired great importance in recent years. Some of the molecules studied include different alkaloids [24,25], coumarins [26], saponins [27], steroids [28] and triterpenes such as betulinic acid [29,30,31]. Derivatives of OA linked to glycosides using click chemistry and esterification methods have recently been reported in the search for inhibitors of glycogen phosphorylase [32]. The aim of the present work was to synthesize some new hybrid compounds between OA alkynyl esters and aromatic azides using click chemistry, developing methodologies that can be applied to other terpenes and natural products as well. The new compounds were assessed for antiproliferative effect using several human tumor cell lines. 

## 2. Results and Discussion

**Scheme 1:**
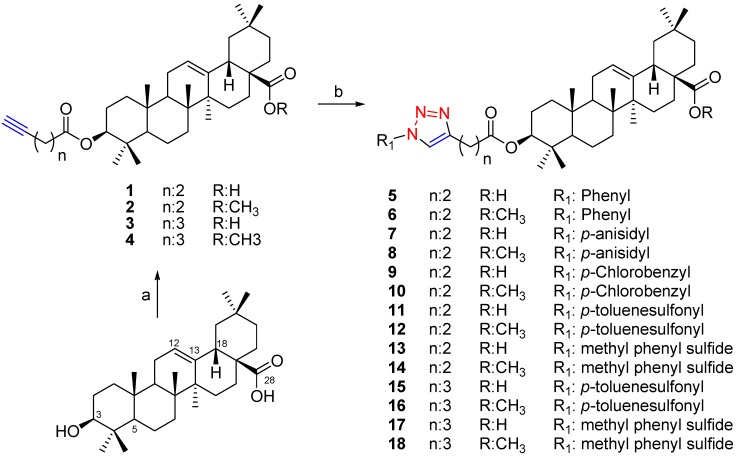
Preparation of oleanolic acid derivatives **1**–**18**.

Starting from the naturally occurring triterpene OA, eighteen derivatives including 14 new hybrid compounds were prepared in moderate to good yields (47–93%) using click chemistry. The compounds were 1,2,3-triazoles linked to the 3-*O* function of OA, either with a free or methylated COOH function at C-28 (Scheme 1). Compounds **1**–**18** are described for the first time. All the products were characterized by spectroscopic means. 

*Reagents and conditions*: (**a**) appropriate alkyne acid, DCC, DMAP, CH_2_Cl_2_, 47–51%; for methyl esters (R:CH_3_) CH_2_N_2_, Et_2_O, 92–93%; (**b**) appropriate aromatic azide, CuSO_4_·5H_2_O, sodium ascorbate, CH_2_Cl_2_:H_2_O 1:1, 47–91%.

The new compounds were assessed for antiproliferative activity towards the following human cell lines: normal lung fibroblasts (MRC-5), gastric epithelial adenocarcinoma (AGS), promyelocytic leukemia (HL-60), lung cancer (SK-MES-1) and bladder carcinoma (J82) cells. IC_50_ values > 100 µM were considered inactive (Table 1). The alkyne esters **1** and **3** showed activity on all cell lines (19.6–23.1 μM and 14.1–56.2 μM, respectively) but without selectivity. Their respective methyl esters **2** and **4** were inactive. Compounds **5**–**8** with a benzene and *p*-anisole attached to the triazole ring, showed no antiproliferative effects. However, introduction of a chlorine atom into the benzene ring (compound **9**) elicited a selective effect against AGS cells (IC_50_ value: 8.9 μM). The activity was lost when the COOH function at C-28 was methylated (compound **10**). Better antiproliferative effect was found for compounds **11** and **15** bearing a *p*-toluenesulphonyl group, with values in the range of 10.8–47.1 μM and 11.5–22.2 μM, respectively. The effect, however, was not associated with selectivity. Compounds **13** and **17** showed similar activity on all cell lines except on MRC-5 cells where compound **13** was inactive and compound **17** showed an IC_50_ value of 46.7 μM. Almost all the methyl esters were inactive with IC_50_ values > 100 µM. The only methyl ester that showed some activity was the compound **12** on HL-60 cells (IC_50_ value: 22.4 μM). This fact shows the important role of the free COOH function at C-28 in OA in the antiproliferative effect.

**Table 1:** Antiproliferative activity of oleanolic acid derivatives **1**–**18** against MRC-5 normal fibroblasts and selected tumor cell lines. *^a^*

**Compound**	**(IC_50_ ± SD, *µ*M) *^b^***				
	**MRC-5**	**AGS**	**SK-MES-1**	**J82**	**HL-60**
**1**	21.3 ± 0.9	19.1 ± 1.0	22.4 ± 1.9	23.1 ± 1.6	19.6 ± 0.1
**2**	>100	>100	>100	>100	>100
**3**	14.1 ± 1.2	22.1 ± 1.5	23.4 ± 1.8	56.2 ± 4.3	22.4 ± 0.1
**4**	>100	>100	>100	>100	>100
**5**	>100	>100	>100	>100	>100
**6**	>100	>100	>100	>100	>100
**7**	>100	95.1 ± 5.8	>100	>100	>100
**8**	>100	>100	>100	>100	>100
**9**	>100	8.9 ± 0.4	50.4 ± 3.5	35.4 ± 2.8	35.8 ± 4.1
**10**	>100	>100	>100	>100	>100
**11**	14.1 ± 0.8	10.8± 0.6	21.4 ± 1.3	47.1 ± 2.9	15.9 ± 1.5
**12**	76.0 ± 3.8	63.0 ± 3.8	76.3 ± 5.1	>100	22.4 ± 1.8
**13**	>100	31.6 ± 1.6	65.5 ± 4.4	>100	18.1 ± 1.6
**14**	>100	>100	>100	>100	>100
**15**	17.1 ± 1.0	22.2 ± 1.6	11.9 ± 0.6	14.3 ± 0.6	11.5 ± 1.0
**16**	>100	>100	>100	>100	84.2 ± 7.5
**17**	46.7 ± 2.4	23.7 ± 1.4	61.8 ± 3.1	>100	11.8 ± 0.9
**18**	>100	>100	>100	>100	>100
Etoposide *^c^*	0.33 ± 0.02	0.58 ± 0.02	1.83 ± 0.09	3.49 ± 0.16	2.23 ± 0.09

*^a^* Cell lines: normal lung fibroblasts (MRC-5), gastric epithelial adenocarcinoma (AGS), promyelocytic leukemia (HL-60), lung cancer (SK-MES-1) and bladder carcinoma (J82) cells. *^b^* Results are expressed as mean values ± SD. Each concentration was tested in sextuplicate together with the control and repeated two times in separate experiments. *^c^* Reference compound.

## 3. Experimental

### 3.1. General Procedures

Melting points were determined on a Koffler hot stage apparatus (Electrothermal 9100, Dubuque, IA, USA) and were uncorrected. Optical rotations were measured on a Jasco DIP 370 (Jasco Analytical Instruments, Easton, MD, USA) polarimeter in CHCl_3_ at 20 °C. IR spectra were recorded on a Nicolet Nexus 470 FT-IR instrument (Thermo Electron Corporation, Waltham, MA, USA). The NMR spectra were recorded on a Bruker Avance 400 (Bruker, Rheinstetten, Germany) spectrometer at 400 MHz for ^1^H and 100 MHz for ^13^C in CDCl_3_. Chemical shifts are given in ppm with TMS as the internal standard. High-resolution mass spectra were measured on a VG Micromass ZAB-2F at 70 eV (Varian Inc., Palo Alto, CA, USA). Merck silica gel (0.063–0.2) was used for column chromatography, pre-coated Si gel plates (Merck, Kieselgel 60 F_254_, 0.25 mm) were used for TLC analysis. TLC spots were visualized by spraying the chromatograms with *p*-anisaldehyde-ethanol-acetic acid-H_2_SO_4_ (2:170:20:10 v/v) and heating at 110 °C for 3 min. Reagents: *N*,*N*-Dicyclohexylcarbodiimide (DCC) and dimethylaminopyridine (DMAP) were from Merck (Schuchardt, Germany). 4-Pentynoic acid, 5-hexynoic acid and aromatic azides were from Aldrich (Schuchardt, Germany). Copper (II) sulphate pentahydrate was from Aldrich (St. Louis, MO, USA) and sodium ascorbate was from Sigma (St. Louis, MO, USA).

### 3.2. General Procedure for the Synthesis of Compounds **1–18**

Oleanolic acid was isolated from the aerial parts of *Fabiana imbricata* as described previously [2] and purified by successive silica gel column chromatography. The compounds **1**–**18** were prepared treating OA with the appropriate alkyne acid/DCC/DMAP to obtain the esters. Treatment with the appropriate azide yielded the corresponding triazole. 

#### 3.2.1. Preparation of Alkynyl Esters **1**, **3**

4-Pentynoic acid or 5-hexynoic acid (1 eq) was dissolved in dry CH_2_Cl_2_ at room temperature under constant stirring. Then, DCC (1 eq) was added, followed after 10 minutes by a catalytic amount of DMAP and OA (1 eq) dissolved in dry CH_2_Cl_2_. The reaction was stopped by adding H_2_O, extracted with CH_2_Cl_2_, dried over Na_2_SO_4_, concentrated and purified. 

#### 3.2.2. General Procedure for the Synthesis of Triazoles **5**, **7**, **9**, **11**, **13**, **15** and **17**

The alkynyl esters **1** or **3** (1 eq) and the corresponding azide (1 eq) were dissolved in CH_2_Cl_2_/H_2_O (1:1), followed by the addition of CuSO_4_.5H_2_O (2 mol%) and sodium ascorbate (10 mol%). The mixture was stirred at room temperature for 24 h. The reaction was stopped by adding H_2_O, extracted with CH_2_Cl_2_, dried over anhydrous Na_2_SO_4_, concentrated and purified by column chromatography on silica gel. 

#### 3.2.3. Preparation of Methyl Esters **2**, **4**, **6**, **8**, **10**, **12**, **14**, **16** and **18**

Methylation was performed using diazomethane in diethyl ether (Et_2_O). Methylation of **1** and **3** yielded the compounds **2** and **4**, respectively. Methylation of the compounds **5**, **7**, **9**, **11**, **13**, **15** and **17** afforded the corresponding methyl esters **6**, **8**, **10**, **12**, **14**, **16** and **18**.

*Compound* (**1**). Oleanolic acid (OA) (170 mg, 0.373 mmol), DCC (77 mg, 0.373 mmol), a catalytic amount of DMAP and 4-pentynoic acid (88 mg, 0.373 mmol), in dry CH_2_Cl_2_ (20 mL), were stirred at room temperature for 2–4 h. The reaction mixture was worked-up as described in 3.2.1. The residue was purified by silica gel column chromatography, eluting with hexane/EtOAc (8:2), yielding **1** (94 mg, 47%): white solid; mp 235 °C; [α]20 *D* +65 (*c* 0.058, CHCl_3_); IR ν_max_ (film) 3309, 2941, 2873, 1731, 1695, 1466, 1270, 760 cm^−1^; ^1^H-NMR (CDCl_3_): δ 5.25 (1H, brs, H-12), 4.52 (1H, t, *J =* 8.3 Hz, H-3α), 2.80 (1H, dd, *J =* 13.5; 3.6 Hz, H-18), 2.48–2.56 (4H, m, OCOCH_2_CH_2_), 1.96 (1H, brs, H-5'), 1.11 (3H, s), 0.92 (3H, s), 0.91 (3H, s), 0.89 (3H, s), 0.86 (3H, s), 0.84 (3H, s), 0.72 (3H, s); ^13^C-NMR (CDCl_3_): δ 184.39 (C-28), 171.52 (C-1'), 143.62 (C-13), 122.49 (C-12), 82.62 (C-4'), 81.33 (C-3), 69.07 (C-5'), 55.27 (C-5), 47.53, 46.53, 45.82, 41.52, 40.87, 39.26, 38.01, 37.73, 36.97, 33.85, 33.78, 33.07, 32.48, 30.66, 29.70, 28.05, 27.66, 25.93, 23.58, 23.53, 23.38, 22.83, 18.15, 17.19, 16.70, 15.36, 14.56; HREIMS *m/z* 536.3768 [M]^+•^ (calcd for C_35_H_52_O_4_, 536.3866). 

*Compound* (**2**). Compound **1** (50 mg, 0.093 mmol), was methylated with a solution of CH_2_N_2_ in ethyl ether, yielding 47 mg (92%) of **2**: white solid; mp 173 °C; [α]20 *D* +71 (*c* 0.046, CHCl_3_); IR ν_max_ (film) 3306, 2945, 2873, 1731, 1461, 1262, 754 cm^−1^; ^1^H-NMR (CDCl_3_): δ 5.25 (1H, brs, H-12), 4.50 (1H, t, *J =* 8.1 Hz, H-3α), 3.59 (3H, s, OMe), 2.82 (1H, dd, *J =* 13.7; 3.8 Hz, H-18), 2.46–2.54 (4H, m, OCOCH_2_CH_2_), 1.94 (1H, brs, H-5'), 1.10 (3H, s), 0.90 (3H, s), 0.89 (3H, s), 0.87 (3H, s), 0.84 (3H, s), 0.83 (3H, s), 0.69 (3H, s); ^13^C-NMR (CDCl_3_): δ 178.23 (C-28), 171.43 (C-1'), 143.78 (C-13), 122.24 (C-12), 82.60 (C-4'), 81.30 (C-3), 69.06 (C-5'), 55.28 (C-5), 51.51 (OMe), 47.52, 46.69, 45.83, 41.62, 41.27, 39.26, 38.06, 37.71, 36.90, 33.83 (2C), 33.11, 32.58, 32.36, 30.68, 28.05, 27.68, 25.90, 23.64, 23.53, 23.39, 23.04, 18.20, 16.82, 16.73, 15.34, 14.51; HREIMS *m/z* 550.4324 [M]^+•^ (calcd for C_36_H_54_O_4_, 550.4022).

*Compound* (**3**). Compound **3** was synthesized as described for compound **1**, using OA and 5-hexynoic acid yielding 105 mg (51%) of **3**: white solid; mp 212 °C; [α]20 *D* +66 (*c* 0.023, CHCl_3_); IR ν_max_ (film) 3308, 2941, 2874, 1729, 1694, 1462, 1276, 757 cm^−1^; ^1^H-NMR (CDCl_3_): δ 5.27 (1H, brs, H-12), 4.50 (1H, t, *J =* 8.3 Hz, H-3α), 2.80 (1H, dd, *J =* 13.4; 3.5 Hz, H-18), 2.45 (2H, t, *J =* 7.5 Hz, H-2'), 2.26 (2H, dt, *J =* 7.0; 2.5 Hz, H-4'), 1.94 (1H, brs, H-6'), 1.83–1.88 (2H, m, H-3'), 1.12 (3H, s), 0.93 (3H, s), 0.92 (3H, s), 0.90 (3H, s), 0.86 (3H, s), 0.85 (3H, s), 0.74 (3H, s); ^13^C-NMR (CDCl_3_): δ 184.01 (C-28), 172.84 (C-1'), 143.59 (C-13), 122.52 (C-12), 83.23 (C-4'), 80.90 (C-3), 69.06 (C-5'), 55.26 (C-5), 47.53, 46.52, 45.81, 41.53, 40.89, 39.25, 38.02, 37.73, 36.97, 33.76, 33.42, 33.05, 32.49, 30.66, 29.69, 28.07, 27.65, 25.91, 23.79, 23.57, 23.38, 22.86, 18.15, 17.91, 17.15, 16.71, 15.36, 14.10; HREIMS *m/z* 550.4410 [M]^+•^ (calcd for C_36_H_54_O_4_, 550.4022).

*Compound* (**4**). Compound **3** (50 mg, 0.089 mmol), was methylated with a solution of CH_2_N_2_ in ethyl ether, yielding 47 mg (93%) of **4**: white solid; mp 176 °C; [α]20 *D* +60 (*c* 0.042, CHCl_3_); IR ν_max_ (film) 3297, 2942, 2868, 1726, 1460, 1226, 754 cm^−1^; ^1^H-NMR (CDCl_3_): δ 5.27 (1H, brs, H-12), 4.50 (1H, t, *J =* 8.1 Hz, H-3α), 3.61 (3H, s, OMe), 2.84 (1H, dd, *J =* 13.8; 3.8 Hz, H-18), 2.44 (2H, t, *J =* 7.5 Hz, H-2'), 2.25 (2H, dt, *J =* 7.0; 2.5 Hz, H-4'), 1.96 (1H, brs, H-6'), 1.81–1.88 (2H, m, H-3'), 1.12 (3H, s), 0.92 (6H, s), 0.89 (3H, s), 0.85 (6H, s), 0.71 (3H, s); ^13^C-NMR (CDCl_3_): δ 178.25 (C-28), 172.78 (C-1'), 143.76 (C-13), 122.22 (C-12), 83.28 (C-4'), 80.87 (C-3), 69.02 (C-5'), 55.24 (C-5), 51.48 (OMe), 47.50, 46.67, 45.80, 41.59, 41.24, 39.24, 38.04, 37.69, 36.88, 33.81, 33.38, 33.06, 32.55, 32.33, 30.65, 28.04, 27.64, 25.86, 23.76, 23.60, 23.51, 23.36, 23.02, 18.17, 17.87, 16.79, 16.71, 15.31; HREIMS *m/z* 564.4164 [M]^+•^ (calcd for C_37_H_56_O_4_, 564.4179).

*Compound* (**5**). Compound **1** (76 mg, 0.142 mmol) and azidobenzene (17 mg, 0.142 mmol), were dissolved in CH_2_Cl_2_/H_2_O (3 mL/3 mL) followed by the addition of 4 mg CuSO_4_.5H_2_O (0.014 mmol, dissolved in 200 *μ*L of water) and 6 mg of sodium ascorbate (0.028 mmol, dissolved in 200 *μ*L of water). The solution was stirred at room temperature for 24 h. The reaction mixture was worked-up as described in 3.2.2 and was purified by silica gel CC eluting with hexane/EtOAc (8:2), yielding **5** (72 mg, 77%). White solid; mp 240 °C; [α]20 *D* +53 (*c* 0.064, CHCl_3_); IR ν_max_ (film) 3415, 2937, 2856, 1727, 1693, 1462, 1277, 761 cm^−1^; ^1^H-NMR (CDCl_3_): δ 7.79 (1H, s, H-5'), 7.68 (2H, d, *J* = 7.8 Hz, H-2'' and H-6''), 7.49 (2H, t, *J* = 7.8 Hz, H-3'' and H-5''), 7.40 (1H, t, *J* = 7.3 Hz, H-4''), 5.25 (1H, brs, H-12), 4.50 (1H, t, *J =* 8.3 Hz, H-3α), 3.12 (2H, t, *J =* 7.2 Hz, H-3'), 2.81 (1H, dd, *J =* 13.4; 3.5 Hz, H-18), 2.79 (2H, t, *J =* 7.0 Hz, H-2'), 1.10 (3H, s), 0.90 (6H, s), 0.88 (3H, s), 0.81 (3H, s), 0.79 (3H, s), 0.72 (3H, s); ^13^C-NMR (CDCl_3_): δ 183.97 (C-28), 172.58 (C-1'), 147.20 (C-4'), 143.68 (C-13), 137.20 (C-1''), 129.72 (2C, C-3'' and C-5''), 128.59 (C-4''), 122.43 (C-12), 120.44 (2C, C-2'' and C-6''), 119.55 (C-5'), 81.26 (C-3), 55.28 (C-5), 47.53, 46.50, 45.86, 41.53, 40.90, 39.25, 38.03, 37.72, 36.95, 34.02, 33.75, 33.08, 32.50, 32.46, 30.68, 27.99, 27.67, 25.92, 23.58, 23.59, 23.38, 22.85, 21.17, 18.15, 17.14, 16.70, 15.36; HREIMS *m/z* 656.4529 [M+H]^+^ (calcd for C_41_H_58_N_3_O_4_, 656.4427). 

*Compound* (**6**). Compound **5** (50 mg, 0.076 mmol), was methylated with a solution of CH_2_N_2_ in ethyl ether, yielding 44 mg (86%) of **6**: white solid; mp 168 °C; [α]20 *D* +44 (*c* 0.050, CHCl_3_); IR ν_max_ (film) 2944, 2876, 1730, 1463, 1260, 756 cm^−1^; ^1^H-NMR (CDCl_3_): δ 7.79 (1H, s, H-5'), 7.69 (2H, d, *J* = 7.7 Hz, H-2'' and H-6''), 7.51 (2H, t, *J* = 7.7 Hz, H-3'' and H-5''), 7.42 (1H, t, *J* = 7.3 Hz, H-4''), 5.27 (1H, brs, H-12), 4.51 (1H, t, *J =* 8.3 Hz, H-3α), 3.62 (3H, s, OMe), 3.13 (2H, t, *J =* 7.2 Hz, H-3'), 2.85 (1H, dd, *J =* 13.4; 3.5 Hz, H-18), 2.80 (2H, t, *J =* 7.0 Hz, H-2'), 1.12 (3H, s), 0.92 (3H, s), 0.91 (3H, s), 0.89 (3H, s), 0.83 (3H, s), 0.80 (3H, s), 0.71 (3H, s); ^13^C-NMR (CDCl_3_): δ 178.35 (C-28), 172.56 (C-1'), 147.24 (C-4'), 143.53 (C-13), 137.56 (C-1''), 129.73 (2C, C-3'' and C-5''), 128.58 (C-4''), 122.26 (C-12), 120.46 (2C, C-2'' and C-6''), 119.50 (C-5'), 81.26 (C-3), 55.30 (C-5), 51.56 (OMe), 47.54, 46.72, 45.84, 41.63, 41.28, 39.27, 38.08, 37.73, 36.91, 34.03, 33.84, 33.11, 32.56, 32.37, 30.70, 27.99, 27.67, 25.90, 23.64, 23.55, 23.40, 23.05, 21.20, 18.19, 16.82, 16.71, 15.34; HREIMS *m/z* 670.5248 [M+H]^+^ (calcd for C_42_H_60_N_3_O_4_, 670.5286). 

*Compound* (**7**). Compound **7** was synthesized as described for compound **5**, using compound **1** (100 mg, 0.187 mmol) and 4-azidoanisole (28 mg, 0.187 mmol) yielding 68 mg (53%) of **3**: white solid; mp 210 °C; [α]20 *D* +35 (*c* 0.045, CHCl_3_); IR ν_max_ (film) 3416, 2946, 2877, 1732, 1695, 1460, 1255, 747 cm^−1^; ^1^H-NMR (CDCl_3_): δ 7.70 (1H, s, H-5'), 7.57 (2H, d, *J* = 8.7 Hz, H-2'' and H-6''), 6.98 (2H, d, *J* = 8.7 Hz, H-3'' and H-5''), 5.25 (1H, brs, H-12), 4.50 (1H, t, *J* *=* 8.3 Hz, H-3α), 3.85 (3H, s, PhOMe), 3.11 (2H, t, *J =* 7.2 Hz, H-3'), 2.80 (1H, dd, *J =* 13.4; 3.5 Hz, H-18), 2.78 (2H, t, *J =* 7.0 Hz, H-2'), 1.11 (3H, s), 0.91 (6H, s), 0.87 (3H, s), 0.82 (3H, s), 0.79 (3H, s), 0.73 (3H, s); ^13^C-NMR (CDCl_3_): δ 183.70 (C-28), 172.60 (C-1'), 159.67 (C-4''), 146.99 (C-4'), 143.68 (C-13), 130.97 (C-1''), 122.45 (C-12), 122.10 (2C, C-2'' and C-6''), 119.75 (C-5'), 114.64 (2C, C-3'' and C-5''), 81.22 (C-3), 55.33 (OMe), 55.25 (C-5), 47.52, 46.50, 45.84, 41.54, 40.92, 39.25, 38.05, 37.73, 36.95, 34.07, 33.77, 33.01, 32.47, 32.30, 30.67, 27.93, 27.69, 25.83, 23.65, 23.53, 23.38, 23.20, 21.18, 18.13, 16.76, 16.64, 15.29; HREIMS *m/z* 686.4309 [M+H]^+^ (calcd for C_42_H_60_N_3_O_5_, 686.4533). 

*Compound* (**8**). Compound **7** (40 mg, 0.058 mmol), was methylated with a solution of CH_2_N_2_ in ethyl ether, yielding 36 mg (89%) of **8**: white solid; mp 151 °C; [α]20 *D* +47 (*c* 0.015, CHCl_3_); IR ν_max_ (film) 2941, 2872, 1722, 1460, 1252, 755 cm^−1^; ^1^H-NMR (CDCl_3_): δ 7.70 (1H, s, H-5'), 7.58 (2H, d, *J* = 8.9 Hz, H-2'' and H-6''), 6.99 (2H, d, *J* = 8.9 Hz, H-3'' and H-5''), 5.27 (1H, brs, H-12), 4.50 (1H, t, *J =* 8.3 Hz, H-3α), 3.86 (3H, s, PhOMe), 3.61 (3H, s, OMe), 3.11 (2H, t, *J =* 7.2 Hz, H-3'), 2.84 (1H, dd, *J =* 13.4; 3.5 Hz, H-18), 2.78 (2H, t, *J =* 7.0 Hz, H-2'), 1.11 (3H, s), 0.91 (6H, s), 0.89 (3H, s), 0.82 (3H, s), 0.79 (3H, s), 0.71 (3H, s); ^13^C-NMR (CDCl_3_): δ 178.26 (C-28), 172.51 (C-1'), 159.63 (C-4''), 146.97 (C-4'), 143.77 (C-13), 130.62 (C-1''), 122.21 (C-12), 122.05 (2C, C-2'' and C-6''), 119.63 (C-5'), 114.68 (2C, C-3'' and C-5''), 81.19 (C-3), 55.58 (PhOMe), 55.27 (C-5), 51.50 (OMe), 47.51, 46.67, 45.81, 41.59, 41.25, 39.24, 38.05, 37.69, 36.89, 34.03, 33.81, 33.07, 32.54, 32.33, 30.66, 27.95, 27.64, 25.86, 23.60, 23.52, 23.36, 23.02, 21.17, 18.16, 16.78, 16.67, 15.31; HREIMS *m/z* 700.4257 [M+H]^+^ (calcd for C_43_H_62_N_3_O_5_, 700.4689). 

*Compound* (**9**). Compound **9** was synthesized as described for compound **5**, using compound **1** (120 mg, 0.224 mmol) and 1-azido-4-chlorobenzene (34 mg, 0.224 mmol) yielding 84 mg (54%) of **9**: white solid; mp 184 °C; [α]20 *D* +46 (*c* 0.052, CHCl_3_); IR ν_max_ (film) 3432, 2942, 2880, 1720, 1688, 1460, 1271, 756 cm^−1^; ^1^H-NMR (CDCl_3_): δ 7.78 (1H, s, H-5'), 7.64 (2H, d, *J* = 8.6 Hz, H-2'' and H-6''), 7.46 (2H, d, *J* = 8.6 Hz, H-3'' and H-5''), 5.25 (1H, brs, H-12), 4.49 (1H, t, *J =* 8.3 Hz, H-3α), 3.13 (2H, t, *J =* 7.0 Hz, H-3'), 2.80 (1H, dd, *J =* 13.4; 3.5 Hz, H-18), 2.78 (2H, t, *J =* 6.9 Hz, H-2'), 1.10 (3H, s), 0.90 (6H, s), 0.88 (3H, s), 0.81 (3H, s), 0.78 (3H, s), 0.72 (3H, s); ^13^C-NMR (CDCl_3_): δ 184.16 (C-28), 172.53 (C-1'), 147.51 (C-4'), 143.65 (C-13), 135.60 (C-1''), 134.28 (C-4''), 129.89 (2C, C-3'' and C-5''), 122.46 (C-12), 121.54 (2C, C-2'' and C-6''), 119.49 (C-5'), 81.29 (C-3), 55.28 (C-5), 47.53, 46.51, 45.85, 41.51, 40.88, 39.24, 38.02, 37.72, 36.96, 33.92, 33.78, 33.08, 32.47, 32.34, 30.68, 27.99, 27.65, 25.92, 23.60, 23.58, 23.39, 22.87, 21.13, 18.21, 17.17, 16.70, 15.36; HREIMS *m/z* 690.4133 [M+H]^+^ (calcd for C_41_H_57_ClN_3_O_4_, 690.4037). 

*Compound* (**10**). Compound **9** (50 mg, 0.073 mmol), was methylated with a solution of CH_2_N_2_ in ethyl ether, yielding 45 mg (88%) of **10**: white solid; mp 206 °C; [α]20 *D* +53 (*c* 0.015, CHCl_3_); IR ν_max_ (film) 2949, 2873, 1725, 1461, 1257, 760 cm^−1^; ^1^H-NMR (CDCl_3_): δ 7.77 (1H, s, H-5'), 7.62 (2H, d, *J* = 8.7 Hz, H-2'' and H-6''), 7.43 (2H, d, *J* = 8.7 Hz, H-3'' and H-5''), 5.23 (1H, brs, H-12), 4.46 (1H, t, *J =* 8.1 Hz, H-3α), 3.58 (3H, s, OMe), 3.08 (2H, t, *J =* 7.2 Hz, H-3'), 2.81 (1H, dd, *J =* 13.6; 3.4 Hz, H-18), 2.75 (2H, t, *J =* 7.2 Hz, H-2'), 1.08 (3H, s), 0.88 (3H, s), 0.87 (3H, s), 0.85 (3H, s), 0.78 (3H, s), 0.75 (3H, s), 0.67 (3H, s); ^13^C-NMR (CDCl_3_): δ 178.21 (C-28), 172.39 (C-1'), 147.42 (C-4'), 143.76 (C-13), 135.59 (C-1''), 134.16 (C-4''), 129.83 (2C, C-3'' and C-5''), 122.20 (C-12), 121.46 (2C, C-2'' and C-6''), 119.40 (C-5'), 81.20 (C-3), 55.26 (C-5), 51.50 (OMe), 47.50, 46.65, 45.80, 41.57, 41.24, 39.22, 38.04, 37.68, 36.87, 33.87, 33.81, 33.11, 32.53, 32.33, 30.66, 27.96, 27.64, 25.88, 23.63, 23.53, 23.36, 23.00, 21.13, 18.16, 16.79, 16.70, 15.32; HREIMS *m/z* 704.4243 [M+H]^+^ (calcd for C_42_H_59_ClN_3_O_4_, 704.4194). 

*Compound* (**11**). Compound **11** was synthesized as described for compound **5**, using compound **1** (120 mg, 0.224 mmol) and *p*-toluenesulfonyl azide (48 mg, 0.224 mmol) yielding 91 mg (55%) of **11**: white solid; mp 163 °C; [α]20 *D* +39 (*c* 0.054, CHCl_3_); IR ν_max_ (film) 3420, 2946, 2871, 1730, 1693, 1460, 1272, 759 cm^−1^; ^1^H-NMR (CDCl_3_): δ 7.96 (2H, d, *J* = 8.3 Hz, H-2'' and H-6''), 7.91 (1H, s, H-5'), 7.36 (2H, d, *J* = 8.3 Hz, H-3'' and H-5''), 5.26 (1H, brs, H-12), 4.47 (1H, t, *J =* 8.3 Hz, H-3α), 3.03 (2H, t, *J* *=* 7.2 Hz, H-3'), 2.81 (1H, dd, *J =* 13.5; 3.1 Hz, H-18), 2.70 (2H, t, *J* *=* 7.2 Hz, H-2'), 2.43 (3H, s, PhMe), 1.11 (3H, s), 0.91 (3H, s), 0.90 (3H, s), 0.89 (3H, s), 0.77 (3H, s), 0.73 (6H, s); ^13^C-NMR (CDCl_3_): δ 184.33 (C-28), 172.05 (C-1'), 147.19 (C-4'), 146.37 (C-4''), 143.64 (C-13), 133.17 (C-1''), 130.43 (2C, C-2'' and C-6''), 128.62 (2C, C-3'' and C-5''), 122.47 (C-12), 121.02 (C-5'), 81.36 (C-3), 55.23 (C-5), 47.53, 46.53, 45.82, 41.51, 40.88, 39.25, 38.03, 37.67, 36.95, 33.78, 33.49, 33.10, 32.46, 32.30, 30.68, 27.95, 27.65, 25.94, 23.60, 23.49, 23.37, 23.14, 21.87, 20.91, 18.13, 17.17, 16.65, 15.37; HREIMS *m/z* 734.4814 [M+H]^+^ (calcd for C_42_H_60_N_3_O_6_S, 734.4203). 

*Compound* (**12**). Compound **11** (60 mg, 0.082 mmol), was methylated with a solution of CH_2_N_2_ in ethyl ether, yielding 53 mg (87%) of **12**: white solid; mp 154 °C; [α]20 *D* +35 (*c* 0.053, CHCl_3_); IR ν_max_ (film) 2946, 2876, 1726, 1467, 1260, 757 cm^−1^; ^1^H-NMR (CDCl_3_): δ 7.94 (2H, d, *J* = 8.3 Hz, H-2'' and H-6''), 7.90 (1H, s, H-5'), 7.34 (2H, d, *J* = 8.3 Hz, H-3'' and H-5''), 5.25 (1H, brs, H-12), 4.44 (1H, t, *J =* 8.2 Hz, H-3α), 3.60 (3H, s, OMe), 3.01 (2H, t, *J =* 7.2 Hz, H-3'), 2.83 (1H, dd, *J =* 13.4; 3.4 Hz, H-18), 2.68 (2H, t, *J =* 7.0 Hz, H-2'), 2.41 (3H, s, PhMe), 1.10 (3H, s), 0.90 (3H, s), 0.87 (6H, s), 0.74 (3H, s), 0.70 (3H, s), 0.69 (3H, s); ^13^C-NMR (CDCl_3_): δ 178.26 (C-28), 171.97 (C-1'), 147.14 (C-4'), 146.36 (C-4''), 143.81 (C-13), 133.19 (C-1''), 130.40 (2C, C-2'' and C-6''), 128.60 (2C, C-3'' and C-5''), 122.21 (C-12), 121.00 (C-5'), 81.31 (C-3), 55.23 (C-5), 51.54 (OMe), 47.51, 46.68, 45.81, 41.60, 41.26, 39.25, 38.03, 37.63, 36.87, 33.83, 33.46, 33.11, 32.54, 32.35, 30.69, 27.91, 27.66, 25.90, 23.64, 23.46, 23.39, 23.03, 21.84, 20.91, 18.17, 16.80, 16.66, 15.33; HREIMS *m/z* 748.4274 [M+H]^+^ (calcd for C_43_H_62_N_3_O_6_S, 748.4359). 

*Compound* (**13**). Compound **13** was synthesized as described for compound **5**, using compound **1 **(120 mg, 0.224 mmol) and azidomethyl phenyl sulfide (37 mg, 0.224 mmol) yielding 98 mg (62%) of **13**: white solid; mp 170 °C; [α]20 *D* +13 (*c* 0.055, CHCl_3_); IR ν_max_ (film) 3426, 2939, 2871, 1723, 1691, 1461, 1276, 756 cm^−1^; ^1^H-NMR (CDCl_3_): δ 7.37 (1H, s, H-5'), 7.29 (5H, s, Ph), 5.57 (2H, s CH_2_S), 5.25 (1H, brs, H-12), 4.47 (1H, t, *J =* 8.3 Hz, H-3α), 3.00 (2H, t, *J =* 7.3 Hz, H-3'), 2.81 (1H, dd, *J =* 13.5; 3.5 Hz, H-18), 2.68 (2H, t, *J =* 7.5 Hz, H-2'), 1.11 (3H, s), 0.91 (3H, s), 0.90 (3H, s), 0.89 (3H, s), 0.80 (3H, s), 0.78 (3H, s), 0.73 (3H, s); ^13^C-NMR (CDCl_3_): δ 184.07 (C-28), 172.42 (C-1'), 147.12 (C-4'), 143.66 (C-13), 132.09 (C-1''), 132.02 (2C, C-2'' and C-6''), 129.46 (2C, C-3'' and C-5''), 128.57 (C-4''), 122.47 (C-12), 120.70 (C-5'), 81.17 (C-3), 55.28 (C-5), 53.65 (CH_2_S), 47.54, 46.52, 45.85, 41.53, 40.89, 39.26, 38.02, 37.71, 36.96, 33.98, 33.79, 33.07, 32.49, 32.44, 30.67, 28.02, 27.66, 25.91, 23.59, 23.52, 23.38, 22.86, 21.19, 18.15, 17.17, 16.70, 15.35; HREIMS *m/z* 702.3102 [M+H]^+^ (calcd for C_42_H_60_N_3_O_4_S, 702.4304). 

*Compound* (**14**). Compound **13** (60 mg, 0.086 mmol), was methylated with a solution of CH_2_N_2_ in ethyl ether, yielding 55 mg (90%) of **14**: white solid; mp 195 °C; [α]20 *D* +17 (*c* 0.102, CHCl_3_); IR ν_max_ (film) 2947, 2875, 1731, 1460, 1257, 763 cm^−1^; ^1^H-NMR (CDCl_3_): δ 7.37 (1H, s, H-5'), 7.28 (5H, s, Ph), 5.56 (2H, s CH_2_S), 5.26 (1H, brs, H-12), 4.46 (1H, t, *J =* 8.1 Hz, H-3α), 3.60 (3H, s, OMe), 2.99 (2H, t, *J =* 7.3 Hz, H-3'), 2.84 (1H, dd, *J =* 13.7; 3.9 Hz, H-18), 2.68 (2H, t, *J =* 7.6 Hz, H-2'), 1.10 (3H, s), 0.90 (3H, s), 0.89 (3H, s), 0.88 (3H, s), 0.80 (3H, s), 0.76 (3H, s), 0.70 (3H, s); ^13^C-NMR (CDCl_3_): δ 178.30 (C-28), 172.39 (C-1'), 147.12 (C-4'), 143.80 (C-13), 132.08 (C-1''), 132.00 (2C, C-2'' and C-6''), 129.45 (2C, C-3'' and C-5''), 128.56 (C-4''), 122.34 (C-12), 120.69 (C-5'), 81.18 (C-3), 55.29 (C-5), 53.63 (CH_2_S), 51.54 (OMe), 47.53, 46.70, 45.83, 41.61, 40.27, 39.25, 38.06, 37.70, 36.90, 33.96, 33.83, 33.11, 32.56, 32.36, 30.69, 28.01, 27.66, 25.90, 23.64, 23.52, 23.39, 23.04, 21.19, 18.18, 16.81, 16.71, 15.33; HREIMS *m/z* 716.4378 [M+H]^+^ (calcd for C_43_H_62_N_3_O_4_S, 716.4461). 

*Compound* (**15**). Compound **15** was synthesized as described for compound **5**, using compound **3 **(120 mg, 0.218 mmol) and *p*-toluenesulfonyl azide (32 mg, 0.218 mmol) yielding 76 mg (47%) of **15**: white solid; mp 186 °C; [α]20 *D* +51 (*c* 0.048, CHCl_3_); IR ν_max_ (film) 3430, 2943, 2873, 1729, 1689, 1461, 1272, 757 cm^−1^; ^1^H-NMR (CDCl_3_): δ 7.94 (2H, d, *J* = 8.2 Hz, H-2'' and H-6''), 7.87 (1H, s, H-5'), 7.34 (2H, d, *J* = 8.1 Hz, H-3'' and H-5''), 5.23 (1H, brs, H-12), 4.47 (1H, t, *J =* 8.1 Hz, H-3α), 2.79 (1H, dd, *J =* 13.4; 3.0 Hz, H-18), 2.73 (2H, t, *J =* 7.5 Hz, H-4'), 2.40 (3H, s, PhMe), 2.32 (2H, t, *J =* 7.3 Hz, H-2'), 1.96 (2H, m, H-3'), 1.09 (3H, s), 0.90 (3H, s), 0.89 (3H, s), 0.87 (3H, s), 0.81 (6H, s), 0.71 (3H, s); ^13^C-NMR (CDCl_3_): δ 184.46 (C-28), 172.82 (C-1'), 147.19 (C-4'), 147.12 (C-4''), 143.63 (C-13), 133.14 (C-1''), 130.44 (2C, C-2'' and C-6''), 128.57 (2C, C-3'' and C-5''), 122.47 (C-12), 120.71 (C-5'), 80.98 (C-3), 55.24 (C-5), 47.51, 46.50, 45.82, 41.48, 40.84, 39.23, 38.03, 37.70, 36.96, 33.81, 33.08, 32.47, 32.33, 31.60, 30.65, 28.10, 27.66, 25.94, 24.65, 24.26, 23.66, 23.59, 23.38, 22.82, 21.84, 18.15, 17.19, 16.75, 15.37; HREIMS *m/z* 748.4327 [M+H]^+^ (calcd for C_43_H_62_N_3_O_6_S, 748.4359). 

*Compound* (**16**). Compound **15** (50 mg, 0.067 mmol), was methylated with a solution of CH_2_N_2_ in ethyl ether, yielding 44 mg (86%) of **16**: white solid; mp 165 °C; [α]20 *D* +32 (*c* 0.079, CHCl_3_); IR ν_max_ (film) 2943, 2871, 1728, 1459, 1254, 757 cm^−1^; ^1^H-NMR (CDCl_3_): δ 7.96 (2H, d, *J* = 8.1 Hz, H-2'' and H-6''), 7.87 (1H, s, H-5'), 7.36 (2H, d, *J* = 8.1 Hz, H-3'' and H-5''), 5.26 (1H, brs, H-12), 4.48 (1H, t, *J =* 8.3 Hz, H-3α), 3.60 (3H, s, OMe), 2.84 (1H, dd, *J =* 13.5; 3.2 Hz, H-18), 2.74 (2H, t, *J =* 7.5 Hz, H-4'), 2.43 (3H, s, PhMe), 2.33 (2H, t, *J =* 7.3 Hz, H-2'), 1.98 (2H, m, H-3'), 1.11 (3H, s), 0.90 (6H, s), 0.88 (3H, s), 0.82 (6H, s), 0.70 (3H, s); ^13^C-NMR (CDCl_3_): δ 178.27 (C-28), 172.74 (C-1'), 147.16 (C-4'), 147.11 (C-4''), 143.80 (C-13), 133.19 (C-1''), 130.42 (2C, C-2'' and C-6''), 128.60 (2C, C-3'' and C-5''), 122.34 (C-12), 120.62 (C-5'), 80.99 (C-3), 55.27 (C-5), 51.55 (OMe), 47.53, 46.69, 45.83, 41.61, 41.27, 39.26, 38.06, 37.70, 36.91, 33.81, 33.11, 32.57, 32.37, 31.58, 30.69, 28.10, 27.66, 25.91, 24.68, 24.27, 23.64, 23.56, 23.39, 23.04, 21.84, 18.20, 16.82, 16.77, 15.35; HREIMS *m/z* 762.4427 [M+H]^+^ (calcd for C_44_H_64_N_3_O_6_S, 762.4516).

*Compound* (**17**). Compound **17** was synthesized as described for compound **5**, using compound **3** (120 mg, 0.218 mmol) and azidomethyl phenyl sulfide (36 mg, 0.218 mmol) yielding 87 mg (56%) of **17**: white solid; mp 198 °C; [α]20 *D* +43 (*c* 0.011, CHCl_3_); IR ν_max_ (film) 3426, 2940, 2876, 1723, 1692, 1461, 1270, 754 cm^−1^; ^1^H-NMR (CDCl_3_): δ 7.30 (1H, s, H-5'), 7.29 (5H, s, Ph), 5.57 (2H, s CH_2_S), 5.25 (1H, brs, H-12), 4.49 (1H, t, *J =* 8.1 Hz, H-3α), 2.80 (1H, dd, *J =* 13.4; 3.2 Hz, H-18), 2.71 (2H, t, *J =* 7.5 Hz, H-4'), 2.31 (2H, t, *J =* 7.3 Hz, H-2'), 1.95 (2H, m, H-3'), 1.11 (3H, s), 0.91 (6H, s), 0.88 (3H, s), 0.84 (3H, s), 0.82 (3H, s), 0.73 (3H, s); ^13^C-NMR (CDCl_3_): δ 184.15 (C-28), 173.06 (C-1'), 147.75 (C-4'), 143.69 (C-13), 132.28 (2C, C-2'' and C-6''), 131.95 (C-1''), 129.47 (2C, C-3'' and C-5''), 128.66 (C-4''), 122.44 (C-12), 120.55 (C-5'), 80.88 (C-3), 55.27 (C-5), 53.72 (CH_2_S), 47.55, 46.52, 45.86, 41.53, 40.89, 39.26, 38.03, 37.73, 36.98, 33.92, 33.76, 33.10, 32.52, 32.41, 30.68, 28.12, 27.67, 25.95, 24.92, 24.73, 23.62, 23.57, 23.41, 22.89, 18.22, 17.18, 16.79, 15.39; HREIMS *m/z* 716.4423 [M+H]^+^ (calcd for C_43_H_62_N_3_O_4_S, 716.4461). 

*Compound* (**18**). Compound **17** (50 mg, 0.068 mmol), was methylated with a solution of CH_2_N_2_ in ethyl ether, yielding 45 mg (91%) of **18**: white solid; mp 166 °C; [α]20 *D* +39 (*c* 0.016, CHCl_3_); IR ν_max_ (film) 2946, 2870, 1723, 1461, 1259, 755 cm^−1^; ^1^H-NMR (CDCl_3_): δ 7.28 (1H, s, H-5'), 7.26 (5H, s, Ph), 5.54 (2H, s CH_2_S), 5.24 (1H, brs, H-12), 4.46 (1H, t, *J =* 8.1 Hz, H-3α), 3.58 (3H, s, OMe), 2.82 (1H, dd, *J =* 13.4; 3.2 Hz, H-18), 2.69 (2H, t, *J =* 7.5 Hz, H-4'), 2.29 (2H, t, *J =* 7.3 Hz, H-2'), 1.93 (2H, m, H-3'), 1.09 (3H, s), 0.88 (6H, s), 0.86 (3H, s), 0.81 (3H, s), 0.80 (3H, s), 0.68 (3H, s); ^13^C-NMR (CDCl_3_): δ 178.23 (C-28), 172.95 (C-1'), 147.71 (C-4'), 143.77 (C-13), 132.24 (2C, C-2'' and C-6''), 131.98 (C-1''), 129.42 (2C, C-3'' and C-5''), 128.61 (C-4''), 122.23 (C-12), 120.48 (C-5'), 80.82 (C-3), 55.26 (C-5), 53.64 (CH_2_S), 51.51 (OMe), 47.51, 46.67, 45.81, 41.59, 41.25, 39.24, 38.06, 37.70, 36.90, 33.89, 33.83, 33.12, 32.56, 32.35, 30.68, 28.10, 27.65, 25.90, 24.93, 24.71, 23.65, 23.57, 23.38, 23.03, 18.19, 16.81 (2C), 15.36; HREIMS *m/z* 730.4572 [M+H]^+^ (calcd for C_44_H_64_N_3_O_4_S, 730.4617). 

### 3.3. Antiproliferative Assay

All human cell lines used in this work were purchased from the American Type Culture Collection (ATCC, Manasas, VA, USA). Normal lung MRC-5 fibroblasts (CCL-171), SK-MES-1 lung cancer cells (HTB-58) and J82 bladder carcinoma cells (HTB-1) were grown as monolayers in minimum essential Eagle medium (MEM) with Earles’s salts, 2 *m*M L-glutamine and 1.5 g/L sodium bicarbonate. Gastric adenocarcinoma AGS cells (CRL-1739) were grown as monolayers in Ham F-12 medium containing 1 mM L-glutamine and 1.5 g/L sodium bicarbonate. Promyelocytic leukemia HL-60 cells (CCL-240) were grown in suspension in RPM1 medium containing 1 mM sodium pyruvate and 2.0 g/L sodium bicarbonate. All media were supplemented with 10% heat-inactivated FBS, 100 IU/mL penicillin and 100 µg/mL streptomycin. Cells were grown in a humidified incubator with 5% CO_2_ in air at 37 °C. For the antiproliferative assay, adherent cells were plated at a density of 5 × 10^4^ cells/mL and HL-60 cells at 30 × 10^4^ cells/mL. Cells were seeded in 96-well plates (100 µL/well). One day after seeding, cells were treated with medium containing the compounds at concentrations ranging from 0 up to 100 µM during 3 days. The compounds were dissolved in DMSO (1% final concentration) and complete medium. Untreated cells (medium containing 1% DMSO) were used as 100% viability controls. Etoposide (98% purity, Sigma-Aldrich, St. Louis, MO, USA) was used as reference compound. Each concentration was tested in sextuplicate and experiments were repeated 2 times. Cell viability was determined by means of the MTT reduction assay at the end of the incubation with the products. The results were transformed to percentage of controls and the IC_50_ values were graphically obtained from the dose-response curves.

## 4. Conclusions

Click chemistry techniques were applied to obtain new heterocycles-terpene hybrid compounds starting from the naturally occurring triterpene OA. The methods presented in this work allowed the synthesis of several new compounds in good to reasonable yields. The compounds were assessed as antiproliferative agents in four human tumor cell lines and on normal fibroblasts. The presence of the free COOH function was relevant for the antiproliferative effect, regardless of the heterocycle moiety. Some of the new products presented better effect than the starting compounds with relative selectivity towards AGS (compound **9**) or HL-60 cancer lines (compound **17**). Additional studies and other assays using different targets are necessary to disclose the potential of the new compounds as bioactive agents. The new hybrid compounds are much less active than the reference compound etoposide and cannot be considered as promising antiproliferative agents. However, other biological activities and mechanisms of action remain to be investigated.
